# Trifolirhizin: A Phytochemical with Multiple Pharmacological Properties

**DOI:** 10.3390/molecules30020383

**Published:** 2025-01-17

**Authors:** Varun Jaiswal, Hae-Jeung Lee

**Affiliations:** 1 Department of Food and Nutrition, College of BioNano Technology, Gachon University, 1342 Seongnam-daero, Sujeong-gu, Seongnam-si 13120, Republic of Korea; computationalvarun@gmail.com; 2 Institute for Aging and Clinical Nutrition Research, Gachon University, Seongnam-si 13120, Republic of Korea; 3 Department of Health Sciences and Technology, GAIHST, Gachon University, Incheon 21999, Republic of Korea; 4 Gachon Biomedical Convergence Institute, Gachon University Gil Medical Center, Incheon 21565, Republic of Korea

**Keywords:** phytochemicals, trifolirhizin, therapeutics, cancer, toxicity, anti-inflammatory

## Abstract

Trifolirhizin is an important flavonoid glycoside reported from the roots of medicinal plants such as Astragalus membranaceus, Sophora tonkinensis, Ononis vaginalis, Euchresta formosana, Sophora Subprostrate, Ononis spinose, and Sophora flavescens. It is considered one of the important constituents responsible for the various medicinal properties of these medicinal plants. Studies have revealed the multiple pharmacological properties of trifolirhizin: anti-inflammatory, antioxidant, antibacterial, anti-ulcerative colitis, antiasthma, hepatoprotective, osteogenic, skin-whitening, wound-healing, and anticancer (against various types of cancers). Mechanistic studies of trifolirhizin showed that it could act on important target genes and pathways such as the NF-κB-MAPK, EGFR-MAPK, AMPK/mTOR, and PI3K/Akt signaling pathways. These pathways are also implicated in various other diseases, suggesting the potential of trifolirhizin in therapeutic applications. Initial pharmacokinetic studies support the therapeutic candidature of trifolirhizin and provide the initial track that may be pursued for its development. Still, a compilation of pharmacological activities and target pathways of trifolirhizin is missing in the literature. This review uniquely compiles the pharmacological properties and mechanistic insights of trifolirhizin, addressing critical gaps in its therapeutic development and proposing strategies for future research.

## 1. Introduction

The advancement of analytical techniques and the pharmacological potential of plant-derived products emphasized the importance of phytocompounds in therapeutics [1]. Among phytocompounds, flavonoid glycosides are known for several significant pharmacological activities [2] that are present in fruits, vegetables, legumes, and other parts of plants. Trifolirhizin is a flavonoid glycoside that is one of the major marker constituents present in the roots of *Sophora flavescens*. Trifolirhizin is also reported in the roots of other plants including *Astragalus membranaceus*, *Sophora tonkinensis*, *Ononis vaginalis, Euchresta formosana, Sophora Subprostrate,* and *Ononis spinose*. The roots of *Sophora flavescens* have been used in traditional Chinese medicine, which is known as Kushen in China. In different studies, numerous pharmacological activities, such as antitumor, anti-inflammatory, antinociceptive, antianaphylaxis, antiasthma, antibacterial, antiviral, insecticidal, antifungal, antiarrhythmic, antimyocardial, vascular relaxing, antimyocardial hypoxic, immunoregulatory, etc., have been reported for *Sophora flavescens* [3]. It is believed that trifolirhizin contributes to the traditional medicinal activities of these plants [4]. Considering the potential role of trifolirhizin in the different pharmacological properties of medicinal plants in which it is present, several pharmacological studies have been conducted to evaluate its potential against different diseases.

**Figure 1:**
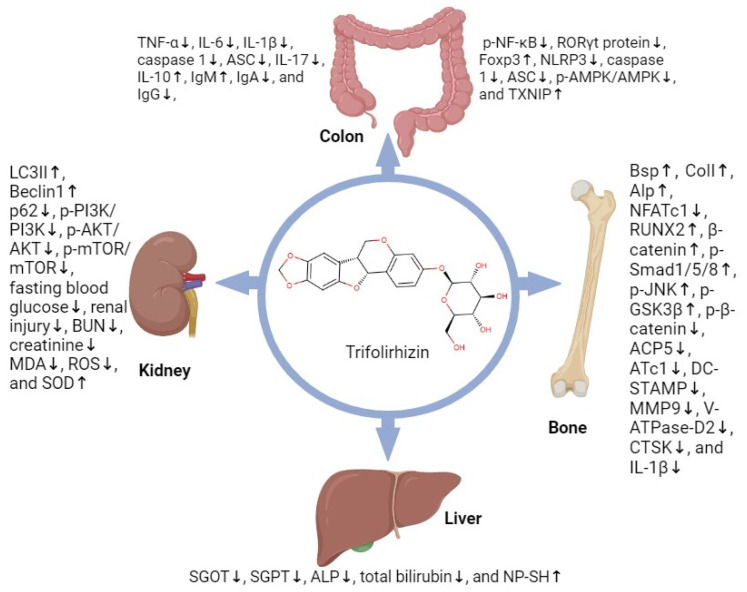
Protective activity of trifolirhizin against different organs. (↑: up-regulation; ↓: down-regulation/inhibition).

Various in vitro, in vivo, and ex vivo studies have highlighted the multiple pharmacological properties of trifolirhizin, such as anti-inflammatory, antioxidant, antibacterial, anti-ulcerative colitis, antiasthma, hepatoprotective, osteogenic, wound-healing, and anticancer activity against lung, gastric, ovarian, nasopharyngeal, hepatocellular carcinoma, and colorectal cancers [5,6,7,8,9,10]. The anti-inflammatory activities of trifolirhizin may contribute to other pharmacological effects, including its anticancer activity [11,12]. The anti-inflammatory activities of trifolirhizin were also found to protect different organs such as the liver, bone, colon, and kidney [13,14,15,16] (Figure 1).

In view of the multiple pharmacological potentials of trifolirhizin, reliable methods have been developed to study the pharmacokinetics of trifolirhizin. The pharmacokinetics of pure trifolirhizin and in a mixture (crude extract) were studied via oral and intravenous administration in rats. These pharmacokinetics studies support the pharmacological potential and can help design doses for oral and intravenous administration in clinical studies [17,18,19]. Research efforts to decipher the molecular mechanism behind important pharmacological activities such as anticancer and bone protection have been conducted in different studies to utilize the optimal potential of trifolirhizin. In these studies, pathways such as the NF-κB-MAPK, EGFR-MAPK, AMPK/mTOR, and PI3K/Akt signaling pathways [6,7,10,13,16] were found to be targeted by trifolirhizin. These important target pathways highlight the pharmacological importance of trifolirhizin and suggest that pharmacological studies of trifolirhizin may be expanded to other important diseases associated with these pathways.

Despite its high pharmacological potential against different diseases and the multiple important targets of trifolirhizin, a compilation of pharmacological activities, target pathways, and genes is still missing in the available literature, which restricts the development of trifolirhizin as a therapeutic/supplement against important diseases such as cancer [7]. Thus, the current review uniquely provides compiled information regarding pharmacological activities, target genes, and pathways, which would be helpful in designing and conducting further research to study the pharmacological activities of trifolirhizin. Importantly, the current review also provides future directions based on the gaps and known targets reported in the literature for the development of trifolirhizin as a candidate against important diseases.

## 2. Electronic Literature Search Strategy

An electronic search for literature was carried out through different databases including ResearchGate, Embase, Scopus, and Web of Sciences. The search included peer-reviewed research articles and review articles published in indexed journals until October 2024. Keywords such as trifolirhizin, pharmacological properties, anticancer, antibacterial, antioxidants, anti-inflammatory, anti-diabetes, anti-ulcerative colitis, antiasthma, hepatoprotective, osteogenic, wound healing, and their combinations were considered in this study.

## 3. Toxicity Studies

Trifolirhizin isolated from the roots of a plant was used in a study, and no toxicity of trifolirhizin was observed at a 50 μM concentration in MTT assays. Similarly, after studying the anti-proliferative activity of trifolirhizin in leukemia cells, researchers found no apoptotic activity on normal lymphocytes prepared from healthy volunteers at a 270–360 μM concentration, which suggests it would not be toxic for healthy tissues of the body [5]. Additionally, the toxicity (IC_50_ > 250 μg/mL) of trifolirhizin was observed against normal kidney and liver cell lines, which supported its safety profile and strengthened its candidature as an anticancer drug [6].

## 4. Pharmacokinetics Studies

Considering the important pharmacological properties of the extract of *Sophora tonkinensis*, a pharmacokinetics study was conducted to analyze important constituents in rat plasma. Trifolirhizin is an important marker compound believed to be essential for different pharmacological activities and was considered in a study along with other phytochemicals. The developed method was based on liquid chromatography with tandem mass spectrometry LC-MS/MS [19]. In the case of trifolirhizin, the developed assay was linear over concentration ranges of 50–5000 ng/mL, with a lower limit of quantification (LLOQ) of 50 ng, which indicated sufficient sensitivity for the investigation of its pharmacokinetic behavior. After a single oral dose (400 mg/kg) of 50% ethanolic extracts of the dried roots of *Sophora tonkinensis* to Sprague Dawley rats, the pharmacokinetic parameters were calculated for trifolirhizin. The area under the plasma concentration–time curve from time zero to last sampling time (AUC_t_), terminal half-life (T_1/2_), peak plasma concentration (C_max_), and time to reach C_max_ (T_max_) were 162 ± 29.0 g min/mL, 107 ± 43.9 min, 1210 ± 386 ng/mL, and 30 (30–90) min, respectively. The validation data, i.e., specificity, precision, accuracy, recovery, and stability, were within the acceptance requirements in the study [19].

In a study, a researcher conducted a pharmacokinetic study of an extract at different doses. In addition to previous studies, the intravenous administration of trifolirhizin was also studied in Sprague Dawley (SD) rats. The intravenous administration of extract at doses of 5, 10, and 20 mg/kg was studied in the rats. After a single dose of 20 mg/kg, pharmacokinetic parameters, such as AUC_t_, T_1/2_, clearance rate (CL), and mean residence time (MRT) values of 42.0 ± 5.31 ng/mL h, 10.6 ± 0.895 min, 22.4 ± 2.42 mL/min/kg, and 8.33 ± 1.22 min, respectively, were observed for trifolirhizin. Importantly, the pharmacokinetics parameters were not significantly different between male and female rats except for T_1/2_, which was higher in female rats [17].

Later, to study the pharmacokinetics of pure trifolirhizin, male SD rats were used [18]. An efficient model to determine trifolirhizin in the plasma of rats was developed through ultra-performance liquid chromatography (UPLC) with Waters (Milford, MA, USA) Acquity UPLC system. In the method, pirfenidone was used as an internal standard, and the developed method had high sample throughput in bioanalysis. After a single oral dose (10 mg/kg) of trifolirhizin to Sprague Dawley rats, the pharmacokinetic parameters of AUC_t_, T_1/2_, C_max_, and mean residence time (MRT) were 552.44 ± 91.26 ng/mL h, 0.68 ± 0.15 h, 1066.83 ± 125.70 ng/mL, and 0.65 ± 0.09 h, respectively [18]. The pharmacokinetics parameters of trifolirhizin in extract and pure form, as obtained through oral and intravenous administrations, would be helpful for the formulation of its dosages in clinical experiments and its further development as a drug candidate.

## 5. Pharmacological Activities of Trifolirhizin

Initially, the primary interest in the pharmacological activities of trifolirhizin was its presence in the roots of *Sophora flavescens* and other medicinal plants [3,20]. Different in vitro, in vivo, and ex vivo studies have shown the role of trifolirhizin against various diseases and conditions. Advanced studies were also conducted to decipher the molecular mechanism behind trifolirhizin’s pharmacological activities. Table 1 summarizes the in vitro properties of trifolirhizin.

### 5.1. Antibacterial Activity of Trifolirhizin

Drug resistance in bacteria is a grave concern for bacterial diseases globally, which demands new antibacterial compounds that can overcome this [23,24]. Natural antibacterial compounds have appeared as a possible answer against drug resistance, which stresses the discovery of new phytochemicals as antibacterial drugs [25,26]. In both in silico and in vitro studies, trifolirhizin has shown strong potential as an antibacterial candidate.

Considering the gastroprotective effects of the trifolirhizin from *Sophora flavescens*, the antibacterial properties of trifolirhizin were studied against *Helicobacter pylori*, which is a pathogenic bacteria involved in liver-associated issues. The antibacterial activity of trifolirhizin was found to be equivalent to the reference antibiotic ampicillin against *H. pylori* at the dose of 100 μg/mL [27].

In an in silico study, Trifolirhizin and Withaperuvin C were selected among 12,000 natural compounds in a series of computational analyses including the Lipinski rule of five, molecular docking against filamenting temperature-sensitive mutant Z protein (FtsZ), pan-assay interference compound (PAINS) filter, absorption, distribution, metabolism, excretion, and toxicity (ADMET) prediction filter, prediction of activity spectra for biologically active substances (PASS) filter, PAINS analysis filter, all-atom molecular dynamic simulations, and principal component and free energy landscape analyses [28]. Finally, the study concluded that trifolirhizin as a lead antibacterial candidate may be pursued for further development after validation through in vitro and in vivo experimentations [28].

### 5.2. Hepatoprotective Activity of Trifolirhizin

Liver diseases are one of the major public health concerns, which cause two million deaths worldwide annually [29]. Liver disease may also increase the risk of various serious diseases of other organs such as diabetes and chronic kidney diseases [29,30]. In view of the anti-inflammatory activity of trifolirhizin, the hepatoprotective property of trifolirhizin isolated from the *Ononis vaginalis* was studied through CCl_4_-induced hepatotoxicity in rats. In the study, Wistar rats were used in three groups, i.e., in treatment, positive control, and negative control groups. Silymarin was administered in the positive control group. The serum levels of transaminase enzymes, such as serum glutamic oxaloacetic transaminase (SGOT) and serum glutamate pyruvate transaminase (SGPT), and alkaline phosphate levels (ALP) were increased in the negative control group due to CCl_4_ toxicity. Similarly, the total bilirubin level in serum was also increased due to jaundice conditions in the negative control group. A significant decrease in all these parameters was observed in the trifolirhizin treatment group, which highlights the hepatoprotective potential of trifolirhizin [31]. Additionally, The level of non-protein sulfhydryl groups (NP-SH) was found to be reduced in the liver due to CCL_4_ toxicity, which was also improved in the trifolirhizin treatment group [31] (Table 2). The study concluded that the hepatoprotective activities of trifolirhizin may be attributed to other properties, such as antioxidant and anti-inflammatory.

### 5.3. Antiplatelet Aggregation Effect of Trifolirhizin

Platelet aggregation drugs are used against important thrombotic diseases, including myocardial infarction and ischemic stroke. The literature has supported the potential use of plant-based therapeutics as antiplatelet aggregation agents [35,36]. In an early study, the antiplatelet aggregation effect of trifolirhizin and macckian isolated from the roots of *Ononis vaginalis* was studied on young female Wistar rats. In the study, blood from Wistar rats was used to study the aggregation of platelets induced by adenosine diphosphate (ADP). In the case of trifolirhizin, the reduction in the aggregation of platelets was found to be 54%, which was much better than that observed in the study for macckian [31] (Table 2).

### 5.4. Estrogenic Effect of Trifolirhizin

In a preliminary study, the estrogenic effect of trifolirhizin and macckian isolated from the roots of *Ononis vaginalis* was studied in young female Wistar rats via the intraperitoneal route [31]. An increase in uterine weight was considered to study the estrogenic effect of the phytocompounds. In the study, treatment, normal control, and positive control (administered the positive drug 17*β*-estradiol) groups were used to analyze the ratios of the weights of the uteri. In comparison, the ratio weight of uteri with whole animals was also studied in control and treated animals. More than a 90% increase in uterine weight was observed in the trifolirhizin-treated group, which was 10 times higher in comparison with the macckian-treated group. It was the first study that showed the effective estrogenic effect of trifolirhizin in the animal model. The study suggested the estrogenic potential of trifolirhizin must be considered in further studies to develop it as a herbal therapeutic (Table 2).

### 5.5. Antiasthma Activity of Trifolirhizin

Asthma is one of the most prevalent non-communicable diseases which affects more than 350 million people worldwide [37]. Traditionally medicines have been used against several diseases including asthma [38]. ASHMI™ (Antiasthma Herbal Medicine Intervention), the extract of *Ganoderma lucidum* (Fr.) P. Karst (Ling Zhi), *Sophora flavescens* Aiton (Ku Shen), and *Glycyrrhiza uralensis* Fisch. ex DC (Gan Cao), has shown antiasthma effects in different in vivo and clinical studies. The inhibition of acetylcholine-induced airway smooth muscle (ASM) contraction in the tracheal rings of allergic asthmatic mice was also observed in a study, but the role of different components and phytochemicals of ASHMI™ was not studied to optimize its effect. Thus, a study was designed to identify the component of the extract and, subsequently, the phytochemical responsible for the inhibition of ASM contraction [4] (Table 2).

Six-week-old BALB/c mice were used to develop an asthmatic mouse model induced by albumin sensitization and challenge. After sacrifice, the tracheas were excised and cut into three tracheal rings, and contraction in tracheal rings was induced by acetylcholine. First, it was found that the extract of *Sophora flavescens*, not *Ganoderma lucidum* or *Glycyrrhiza uralensis*, inhibited ASM contraction in tracheal rings of the asthmatic mice induced by acetylcholine. Further, with the use of preparatory HPLC, the bioassay-guided fractionation was conducted to identify flavonoid compounds in *S. flavescens* that inhibit ASM contraction. Finally, trifolirhizin was identified as the constituent responsible for the suppression of ASM contraction with the help of preparative HPLC fractionation, LC-MS, and NMR spectroscopy. Trifolirhizin significantly suppressed the acetylcholine-induced contraction at 6.0 μg/mL [4]. The study suggested further mechanistic study may be conducted to develop trifolirhizin as a new candidate for asthma by modulating ASM contraction.

Later, the antiasthma activity of trifolirhizin was studied on neonatal Sprague Dawley rats littered from pregnant female rats [34]. Ovalbumin was used to induce tissue damage and inflammation in the lungs, and three treatment doses (2, 4, and 5 mg/kg) along with negative control (asthma group) and sham groups were used in the study. The serum IgE was dose-dependently reduced in the treatment group but was increased in the negative control group as compared to the sham group. Similarly, significant dose-dependent suppression of histological scores was observed with the treatment, which was increased in the negative control group as tissue damage, aggregation of inflammatory cells, and edema in pulmonary tissues were seen in the negative control group. Similarly, the expression of the *Muc5AC* and *Muc5B* genes in the lungs and the levels of TNF-α and ICAM-1, IL-4, IL-5, and IL-13 in BALF were suppressed in a dose-dependent manner in the treatment groups, which were increased in the asthma group. The WB analysis showed the upregulation of IκBα protein expression in the treatment groups, which was suppressed in the asthma group. The upregulation of the IκBα protein indicated the antiasthma activity of trifolirhizin may be associated with the regulation of the NF-κB signaling pathway [34].

### 5.6. Anti-Inflammatory Effect of Trifolirhizin

Various phytochemicals from numerous plants are known for their anti-inflammatory activities [39,40], which are believed to be one of the important factors responsible for their pharmacological properties [39,40]. The anti-inflammatory activity of trifolirhizin was studied in different in vitro and in vivo studies. The anti-inflammatory activity of trifolirhizin can be considered an important activity as it was also found to support other important activities of trifolirhizin such as anti-UC and bone protection; thus, anti-inflammatory activities were also analyzed in anti-UC and bone protection activities. The anti-inflammatory activity regarding anti-UC and bone protection activities were discussed in the respective sections.

#### 5.6.1. In Vitro Anti-Inflammatory Effect of Trifolirhizin

In a study, the anti-inflammatory activity of trifolirhizin was analyzed through lipopolysaccharide (LPS)-treated J774A.1 macrophages of the mouse [8]. In real-time quantitative PCR analysis, the mRNA expression of pro-inflammatory cytokines, such as tumor necrosis factor (TNF-α) and interleukin-6 (IL-6), was found to be significantly decreased with trifolirhizin treatment, which increased due to the LPS application. The expression of TNF-α was also found to be suppressed in an ELISA study. The suppression of TNF-α and IL-6 by trifolirhizin treatment was also observed in other studies [33,34]. Similarly, the protein expression of cyclooxygenase-2 (COX-2) was also found to be significantly decreased with trifolirhizin treatment, which increased by LPS application [8] (Table 1).

#### 5.6.2. In Vivo Anti-Inflammatory Effect of Trifolirhizin

In a study, the anti-inflammatory activity of trifolirhizin was analyzed through the carrageenan-induced hind paw edema model in mice. In an in vivo experiment, treatment, normal control, and positive control (indomethacin was used as a positive drug) groups were studied. In the study, trifolirhizin was found to be less active as compared to indomethacin. The reduction in edema by trifolirhizin was only 35.5%, which was insignificant in the study [31] (Table 2).

### 5.7. Anti-Ulcerative Colitis Activity of Trifolirhizin

Recently, studies have strengthened the candidature of plants and their phytochemicals as therapeutics against ulcerative colitis, a chronic inflammatory bowel disease [41,42,43]. The anti-inflammatory activities of trifolirhizin, validated through both in vitro and animal studies, hint at the possible activity of trifolirhizin against ulcerative colitis (UC), which is a chronic inflammatory disease. A research group conducted an animal study on C57BL/6 mice to evaluate the anti-UC activity of trifolirhizin [33]. Dextran sulfate sodium (DSS) was used to induce UC in model mice for the evaluation of (12.5, 25, and 50 mg/kg) doses of trifolirhizin. In the study, the disease activity index (DAI) was significantly inhibited in all treatment groups and was increased in the DSS group. Similarly, DSS induced the shortening of colon length, which was also significantly improved in the 25 and 50 mg/kg trifolirhizin treatment groups. The weight of mice was also improved in a dose-dependent manner, which was reduced significantly in the DSS group. The suppression of anti-inflammatory genes/markers, such as TNF-α, IL-6, and IL-1β, was also studied in the DSS and treatment groups to study the role of inflammation factors in the treatment. The mRNA expression of TNF-α, IL-6, and IL-1β was found to be suppressed by the trifolirhizin in the quantitative reverse transcription (qRT)-polymerase chain reaction (PCR). The ELISA analysis also revealed that the protein expression of TNF-α, IL-6, and IL-1β was found to be suppressed by trifolirhizin in a dose-dependent manner. Further, a Western blot analysis revealed that the protein expression of p-nuclear factor (NF)-κB/NF-κB was suppressed in the treatment groups, which was increased in the DSS group. The study suggested that trifolirhizin may reduce inflammation by targeting the NF-κB pathway [33].

Flow cytometry analysis was used to study the Th17/Treg balance in cells from mesenteric lymph nodes and the spleen. In the DSS group, an increase in the proportion of Th17 (CD4^+^ IL17^+^) cells was observed, which was suppressed by trifolirhizin in a dose-dependent manner. Conversely, the proportion of Treg (CD4^+^ CD25^+^ Foxp3^+^) cells was enhanced by the trifolirhizin treatment in a dose-dependent manner. Subsequently, the levels of IL-17 and IL-10 were found to be decreased and increased, respectively, in the trifolirhizin treatment groups, which were found to be increased in the DSS group. Further, the expression of the RORγt and Foxp3 proteins was found to be suppressed and enhanced, respectively, in the trifolirhizin treatment groups. Additionally, the trifolirhizin treatment suppressed the increase in the level of the IgA and IgG proteins induced by DSS and enhanced the expression of IgM, which was also elevated in the DSS group. Overall, the findings suggested that trifolirhizin regulated the Th17/Treg balance in DSS-induced UC mice. The effect of trifolirhizin was also studied on the NLRP3 inflammasome in the DSS-induced UC mice; the gene expressions of the NLRP3, caspase 1, and ASC genes were found to be suppressed in the treatment groups, which were increased in the DSS group. Subsequently, the protein expressions NLRP3, caspase 1, ASC, IL-1β, and caspase 1 were also found to be suppressed in the trifolirhizin treatment groups, which were increased in the DSS group. Additionally, using immune staining experiments, NLRP3 expression was also found to be downregulated in the trifolirhizin treatment groups, which was increased in the DSS group of mice. The results indicated the suppression of the stimulation of the NLRP3 inflammasome in DSS-induced UC mice [33].

Furthermore, the expression of phosphorylated AMP-activated protein kinase (p-AMPK) and thioredoxin-interacting protein (TXNIP) was decreased and increased, respectively, in the DSS group. However, the reverse effect on the expression of these proteins was observed in the trifolirhizin treatment groups. The study not only revealed the effective anti-UC activities of trifolirhizin but also provided the leading role of anti-inflammation activity to support it. Overall, the study suggested that trifolirhizin may exert its anti-inflammatory effects by regulating the balance of Th17/Treg cells and the suppression of the NLRP3 inflammasome by targeting the AMPK-TXNIP pathway. The study strongly recommended the further validation of findings on clinical samples for the development of trifolirhizin as a candidate against UC [33].

### 5.8. Protective Effect of Trifolirhizin on Bone

Osteoporosis (bone loss) is a serious health concern worldwide, which can be accrued due to several reasons, and elderly people are more affected by it [44]. The protective effect of trifolirhizin on bones was studied in in vitro and in vivo studies through different models [15].

#### 5.8.1. In Vitro Protective Effect of Trifolirhizin on Bone

Given the different biological properties of *Sophora flavescens,* trifolirhizin isolated from *Sophora flavescens* was studied for a positive effect on osteoblast differentiation followed by intracellular signaling. MC3T3-E1 cells were used to study osteogenesis in the study. Trifolirhizin isolated from the roots of the plant was used in the study, and no toxicity of trifolirhizin was observed at a 50 μM concentration in MTT assays. The effect on osteogenic activity was studied through alkaline phosphatase activity, which was found to be increased in trifolirhizin-treated cells through activity and staining assays. Further, the expression levels of important osteogenesis marker genes, such as collagen type I (ColI), bone sialoprotein (Bsp), and Alp, were also found to be enhanced in the trifolirhizin-treated cells. Additionally, changes associated with bone formation, including morphological changes, migration, and deposition of calcium, were studied through the detection of F-actin polymerization in the cells, cell migration through Matrigel-coated membranes, and ARS staining to study the mineralization by calcium deposits in the extracellular matrix, respectively. All these changes were found to increase with trifolirhizin treatment, which supports its osteogenic effects. Finally, the molecular mechanism underlying the osteogenic effects of trifolirhizin was also studied, and the expression of RUNX2, the transcription factor known to stimulate the expression of osteogenesis genes such as Alp, ColI, and Bsp, was found to be significantly increased in Western blot experiments. The accumulation of RUNX2 was also found to be increased in the nucleus region through a marker in the immunofluorescence assay. The signaling involved in osteogenesis through RUNX2 expression was also studied. The study suggested that trifolirhizin regulates major osteogenic signaling proteins, including p-GSK3β, β-catenin, p-Smad1/5/8, and p-JNK, by stimulating the phosphorylation of Smad1/5/8, JNK, and GSK3β, and increases the expression and dephosphorylation of β-catenin [15] (Table 1). In a recent study, osteoclast differentiation was also studied through bone marrow macrophage (BMM) extracted from the bone marrow cavity of the femur and tibia of C57BL/6J mice. Receptor activator of nuclear factor-κB ligand (RANKL) was used to stimulate differentiation in the study as RANKL-induced MAPK-NFATc1 signaling pathways stimulate the expression of NFATc1 protein for osteoclast formation. A significant reduction in the formation of multinucleated osteoclasts was observed at all doses (10, 20, and 40 μM) used in the study. Additionally, the marker genes involved in osteoclast formation, such as *ACP5*, *ATc1*, *DC-STAMP*, *MMP9*, *V-ATPase-D2*, and *CTSK*, were found to be downregulated in a dose-dependent manner in the treatment groups. In a bone resorption analysis, trifolirhizin treatment (at 20 and 40 μM) significantly decreased absorption area. The study showed that trifolirhizin has the potential to impede osteoclast formation and hinder bone resorption abilities. Regarding osteoclast differentiation, trifolirhizin also reduced the protein expression of the NFATc1 protein in a Western blot analysis, which suggested that trifolirhizin inhibits osteoclast formation by targeting ANKL-induced MAPK-NFATc1 signaling pathways [14]. Later, the researchers found a dose-dependent inhibition of osteoclast differentiation without affecting the cell proliferation of BMM cells activated through RANKL [13]. The morphological changes associated with osteoclast differentiation such as actin ring formation were found to be inhibited, as observed by using rhodamine–phalloidin staining in trifolirhizin treatment.

Similarly, trifolirhizin treatment resulted in the inhibition of osteoclast acidification and the dose-dependent suppression of bone resorption. Additionally, the expression of osteoclast-associated genes, such as Fos, Nfatc1, Atp6v0d2, Dcstamp, Acp5, and Ctsk, was downregulated by trifolirhizin, which was upregulated by RANKL. Further, the role of trifolirhizin treatment on NF-κB and MAPK signaling pathways was also studied. The translocation of P65 into the nucleus from the cytoplasm was reduced by trifolirhizin treatment and was observed through nuclear translocation immunofluorescence assays. The phosphorylation of P65 and the degradation of the IκBα protein were suppressed by trifolirhizin treatment, which was stimulated by RANKL. Regarding the MAPK signaling pathway, trifolirhizin treatment decreased the phosphorylation of ERK and JNK phosphorylation primarily at 10 min and 10–20 min of RANKL stimulation. Trifolirhizin treatment also prevented the NFATc1 nuclear translocation and protein expression of NFATc1, ATP6V0D2, and CTSK [13].

Trifolirhizin treatment also significantly reduced the reactive oxygen species production during the osteoclast differentiation stimulated by RANKL, as observed through a cell-permeable fluorescent probe (H2DCFDA)-based assay. Further study revealed that the gene expression of the Nfe2l2 and Hmox1 genes and the antioxidant enzymes HO-1 and CAT were enhanced with trifolirhizin treatment, which were suppressed by RANKL.

The role of inflammation was also studied on BMM cells induced through LPS through the expression of inflammation-related factors such as TNF-α and IL-1β. The gene expression of TNF-α and IL-1β was found to be suppressed, which was stimulated by LPS. Similarly, the protein expression of TNF-α was found to be suppressed with trifolirhizin treatment [13].

#### 5.8.2. In Vivo Protective Effect of Trifolirhizin on Bone

In view of the osteogenic activities of trifolirhizin through in vitro cell line analysis and the downregulation of marker genes of osteoclast formation, a study was conducted to analyze the effect of trifolirhizin on bone loss in mice [14] (Table 2). An ovariectomized (OVX) mice model was used in the study to analyze the effect of trifolirhizin (20 and 40 mg/kg doses) on ovariectomy-induced bone loss in the mice in 6 weeks. In a μCT scanning analysis of the tibia, the extensive bone loss due to ovariectomy in the mice was significantly recovered in the high-dose-treated group. In a histological study, TRAP-positive osteoclasts were also reduced in the trifolirhizin group compared with the ovariectomized group, which had a clear trabecular reduction and bone loss. The entire study suggested that trifolirhizin can target the MAPK-NFATc1 pathway and associated marker genes to inhibit osteoclast production and bone resorption activity, which may result in the recovery of bone loss [14].

The activity of trifolirhizin against osteolysis caused by LPS-induced inflammation was studied in an in vivo model of mice. Two doses of trifolirhizin (5 and 10 mg/kg) were used to evaluate its effect on osteolysis induced through LPS locally on the cranial cap bone of mice. Trifolirhizin treatment significantly improved osteolysis in a dose-dependent manner. Similarly, bone volume/tissue volume (BV/TV) and bone destruction area were also improved in a dose-dependent manner as compared to the LPS group of animals. The bone protective effect of trifolirhizin and reduction in the number of multinucleated TRAP-positive osteoclasts was also observed in staining experiments. The inflammatory factor IL-1β-positive area in bone was also reduced with trifolirhizin treatment, as observed in an immunohistochemistry analysis [13]. Although the animal models and provided doses were different in both in vivo studies, a reduction in TRAP-positive osteoclasts and effective protection against bone loss were observed in both of the studies. It can be concluded that trifolirhizin may develop as a therapeutic against bone loss due to different conditions.

### 5.9. Skin-Whitening and Tyrosinase Inhibition Activity

Tyrosinase is a multifunctional enzyme that is useful in various industries such as food, agriculture, medicine, and cosmetics. Tyrosinase involves melanin synthesis, which is responsible for pigmentation; thus, tyrosinase inhibitors are used as a skin-whitening agent in cosmetics [45].

The high tyrosinase inhibitory activity of the extract of *Sophora flavescens*, in comparison with some known constituents of the extract, suggested studying the tyrosinase inhibitory activity of other constituents of the extract including trifolirhizin [20]. First, the methanolic extract and its different fractions were studied for tyrosinase inhibition activity. The ethyl acetate fraction was found to have the highest activity as compared with the methanolic extract and other fractions of the extract. Thus, the ethyl acetate fraction was used to study the phytochemicals responsible for tyrosinase activity. Three compounds including trifolirhizin were studied for tyrosinase activity. The IC_50_ of trifolirhizin was found to be 506.77 μM in the enzyme inhibition assay. Additionally, to study the effect of selected phytochemicals on melanogenesis in melanocytes for skin-whitening potential, B16 melanoma cells were used. Trifolirhizin was able to reduce the production of intracellular melanin by 76% (at a 50μM concentration), which was increased due to the 3-isobutyl-1-methylxanthine (IBMX) inducer used in the study. The study highlighted the skin-whitening potential of trifolirhizin along with other studied phytochemicals [20] (Table 1).

### 5.10. Anti-Diabetic Nephropathy

Diabetic nephropathy is a diabetic kidney disease that is considered one of the main causes of end-stage renal diseases [46]. The increase in the occurrence of diabetes worldwide has also raised an important concern and complication for diabetic nephropathy [47,48]. Herbal medicines have shown therapeutic potential against diabetes and associated conditions [43,49,50].

The effect of trifolirhizin against diabetic nephropathy was studied on a male db/db mice model to determine its effect on renal injury [16] (Table 2). Trifolirhizin treatment reduced both the body and renal weight of diabetic mice, which increased in comparison with the control group. Trifolirhizin treatment also reduced the increase in urine and fasting blood glucose levels due to diabetes. The renal injury-related parameters, such as glomerular hypertrophy, tubular basement membrane thickening, and mesangial matrix expansion, observed in the diabetic mice were improved with trifolirhizin treatment. Similarly, serum creatinine and blood urea nitrogen (BUN) were increased in the diabetic model, but trifolirhizin treatment reduced both of these parameters. These results highlighted the renal protective activity of trifolirhizin. Further, a TUNEL assay revealed that trifolirhizin suppressed apoptosis in the renal tissues of the diabetic mice and activated autophagy by enhancing the expression of LC3II and Beclin1 and suppressing the expression of p62 observed in the WB analysis. The antioxidant effect of trifolirhizin was studied by analyzing the level of malondialdehyde (MDA) and superoxide dismutase (SOD). Trifolirhizin treatment decreased MDA and lactate dehydrogenase (LDH) levels, which were increased in the diabetic mice. Conversely, trifolirhizin treatment increased the SOD level, which was decreased in the diabetic mice. In the dihydroethidium (DHE) staining, trifolirhizin treatment also suppressed the ROS level, which was increased in the diabetic mice. Studies suggested that the antioxidant activity of trifolirhizin may also contribute to its protective effects [51]. In renal tissues, the activation of the PI3K/AKT/mTOR pathway was observed in diabetic mice through an increase in the phosphorylation levels of, AKT, mTOR, and PI3K. Trifolirhizin treatment decreased the levels of p-AKT, p-mTOR, and p-PI3K, which suggested that trifolirhizin targets the PI3K/AKT/mTOR pathway to alleviate injury in renal tissues [16]. Suppression of the mTOR signaling pathway was also observed in the study, deciphering the molecular mechanisms of the anticancer activity of trifolirhizin [7].

### 5.11. Anticancer Effects of Trifolirhizin

Generally, phytochemicals are considered important candidates against cancer compared to synthetic compounds due to low toxicity [52,53]. Numerous phytochemicals are reported as anticancer candidates in the literature, which also supports the anticancer activity of plant products [54,55].

#### 5.11.1. In Vitro Anticancer Activities of Trifolirhizin

In an initial study, the anticancer activity of phytochemicals trifolirhizin and maackiain isolated from *Sophora subprostrate* were studied for anticancer activities against human leukemia cells (HL-60 cells). Trifolirhizin significantly inhibited the proliferation of HL-60 cells in a dose-dependent manner [5]. The occurrence of apoptosis through morphological changes, such as apoptotic bodies and fragmentation of genomic DNA, was also observed in the treated cells. Further, a mechanistic study indicated the role of active oxygen in the anti-proliferative effect of trifolirhizin instead of the caspase cascade [5] (Table 1). Furthermore, researchers found no apoptotic activity of trifolirhizin on normal lymphocytes prepared from healthy volunteers at a 270–360 μM concentration, which suggests it would not be toxic to healthy tissues of the body.

Later, inspired by the earlier anticancer activity of trifolirhizin, it was isolated from the roots of *Sophora flavescens* and studied for anti-proliferative activities against A2780 ovarian cancer and H23 lung cancer cell lines. The significant anti-proliferative activity of trifolirhizin was observed on both the cell lines but was higher in the case of A2780 ovarian cancer cells as compared to H23 lung cancer cells. The higher anti-proliferative activity of trifolirhizin indicated the potential selectivity of trifolirhizin as an anticancer agent [8] (Table 1). The study suggested that the anti-proliferative activity may be due to the anti-inflammatory mechanism of trifolirhizin, which was confirmed by the suppression of inflammatory genes/markers, as observed through the real-time quantitative polymerase chain reaction (RT-QPCR) and Western blot experiments conducted in the study [8].

Later, the anticancer activity of trifolirhizin was studied through anti-proliferation effects on MKN45 cells (human gastric cancer cells) by an MTT assay. In the experiment, the time- and dose-dependent inhibition of MKN45 cells was observed. Additionally, cell shrinkage, chromatin compaction of apoptosis, nuclear fragmentation, and an increase in the number of apoptotic cells were observed in the treated cells. However, the limited anti-proliferative activity (IC_50_ > 250 μg/mL) of trifolirhizin was observed against normal kidney and liver cell lines, which supported its safety profile and strengthened its candidature as an anticancer drug. In a Western blot analysis, cyclin B, Cdc2 p-EGFR, p-ERC, and p-MEK were downregulated and p-P38 was upregulated with trifolirhizin treatment. Further, the stimulation of c-Myc, caspase-9, and caspase-3 were also observed in the study [6] (Table 1). Trifolirhizin also caused P53 activation, which may be the important reason for cell cycle arrest observed through accumulation in the G2/M phase in MKN45 cells with treatment.

The anti-proliferative activities of trifolirhizin against different cancer cell lines and the limited knowledge of action mechanisms inspired researchers to study the signaling behind its anticancer activity. Two cell lines of colorectal cancer, HCT116 and SW620 cells, were used in the study [7]. Trifolirhizin significantly inhibited the cell viability of both HCT116 and SW620 cells in a dose-dependent manner, as observed through a CCK-8 assay. Initially, autophagy was observed through marker proteins LC-3 and p62/SQSTM-1 in ab immuno-blotting assay with trifolirhizin treatment. Additionally, autophagic vacuoles were also visualized in the trifolirhizin treatment cells through transmission electron microscopy. Trifolirhizin treatment also increased the autophagosomes and autophagolysosomes, as observed through an AdmCherry-GFP-LC3B fluorescent assay, which showed an enhanced autophagy flux in colorectal cancer, HCT116, and SW620 cells. Further, autophagy inhibitors were used to verify the autophagy flux stimulated with trifolirhizin. The addition of different autophagy inhibitors resulted in the impairment of autophagy flux at different degrees [7] (Table 1).

The adenosine monophosphate-activated protein kinase (AMPK)/mammalian target of the rapamycin (mTOR) signaling pathway is considered an important pathway for autophagy and was studied for trifolirhizin action. Trifolirhizin treatment promoted the phosphorylation of AMPK and inhibited the phosphorylating of mTOR.

The AMPK-specific inhibitor or small interfering RNA (siRNA) resulted in the reversed manner of trifolirhizin action, i.e., the inhibitor or siRNA inhibited the phosphorylation of AMPK and the dephosphorylation of mTOR, which suggested that AMPK activation was essential for trifolirhizin-induced autophagy. While rapamycin, an mTOR inhibitor, acted differently, which enhanced the induction of autophagy caused by trifolirhizin [7]. Further, after studying the short- and long-term growth inhibitory effect of trifolirhizin in both HCT116 and SW620 cells through CCK-8 and colony formation assays [7], early and late apoptosis in both HCT116 and SW620 cells were observed through flow cytometry analysis and TUNEL staining. The intrinsic pathway of apoptosis may not be involved in trifolirhizin-mediated apoptosis, as the concentrations of cytochrome c and cleaved caspase-9 were not altered with trifolirhizin treatment. However, the extrinsic pathway of apoptosis may be associated with trifolirhizin, as an enhancement in cleaved caspase-3, cleaved caspase-8, and cleaved poly ADP-ribose polymerase was observed with the treatment. Further, the administration of Z-VAD-FMK, a pan-caspase inhibitor, reduced the cytotoxicity of cells caused by trifolirhizin, suggesting that the caspase cascade may be an important mechanism associated with the trifolirhizin-induced apoptosis that occurred in the cells. Additionally, inhibitors of AMPK signaling, early autophagy, and late autophagy, such as ATG5 siRNA, compound C, and CQ, respectively, were used to study their effects on trifolirhizin treatment. These inhibitors reversed the trifolirhizin-mediated apoptosis [7] (Table 1).

A study was conducted to evaluate the synergistic anticancer effect of anticancer phytochemicals from the medicinal plant *Sophora alopecuroides* with the anticancer drug sorafenib. Sorafenib is an FDA-approved drug used for advanced hepatocellular carcinoma, but toxicity issues at high doses limit its usage [56]. Hepatocellular carcinoma cell lines (HCCCLs), such as MHCC97H, MHCC97L, and HepG2, were used to study synergistic anticancer activity [9]. In the study, the highest synergistic anticancer activity of trifolirhizin, which was 3.5 times better than the sorafenib alone, was observed against the MHCC97H cell line (Table 1). A significant increase in nucleus fragmentation, apoptotic body formation, and percentage of apoptotic cells by 5 times more than that with sorafenib alone was observed in the MHCC97H cell line through the DAPI staining assay. Similarly, MHCC97H cells arrested more in the G1 phase than with sorafenib alone, as observed through a cell cycle assay, and the inhibition of cyclin D1 and cyclin B1 was also revealed in a WB analysis, which supports the inhibition of the G1 phase.

The mitochondrial role in apoptosis was also studied through the JC-1 assay of MHCC97H cells, which revealed the depletion of mitochondrial membrane potential MMP (ΔΨm), supporting the mitochondrial apoptotic pathway. Further, the increased expression of cleaved caspase-9 and caspase-3 treated by a combination was observed compared to either agent alone. Additionally, Bax/Bcl-2 expression was also found to be increased, which again suggested the role of the mitochondrial apoptotic pathway in the apoptotic effect of the synergistic action of the treatment. MAPK signaling was also studied for the action of combination, and, again, a reduction in JNK and P38 expression levels compared to either agent alone was observed in the MHCC97H cells. Similarly, the expression levels of p-AKT and p-ERK reduced significantly compared to either agent alone, which suggested the targeting of the ERK and AKT signaling pathways by the combination of trifolirhizin and sorafenib [9] (Table 1).

Recently, the anticancer activity of trifolirhizin was studied in nasopharyngeal carcinoma. The NP69 nasopharyngeal epithelial cell line and human nasopharyngeal carcinoma cell lines (6–10 B and HK1) were used to analyze the anticancer potential of trifolirhizin. In CCK-8 assays, the viability of both nasopharyngeal carcinoma cell lines was decreased in a time- and dose-dependent manner. The same concertation of trifolirhizin was less toxic for the normal nasopharyngeal epithelial cell line NP69 [10].

Further, a flow cytometry analysis through PI staining revealed that trifolirhizin arrested nasopharyngeal cancer cells at the G0/G1 phase. Significant anti-migration and anti-invasion activities of trifolirhizin were also observed on both nasopharyngeal carcinoma cell lines. In a scratch assay, a significant reduction in 6–10 B and HK1 cell migration was observed in trifolirhizin-treated cells as compared to control cells. Similarly, in Transwell assays, a significant reduction in invaded cell numbers in both 6–10 B and HK1 cells was observed in a dose-dependent manner, which suggested a strong anti-invasion activity of the trifolirhizin [10].

**Figure 2:**
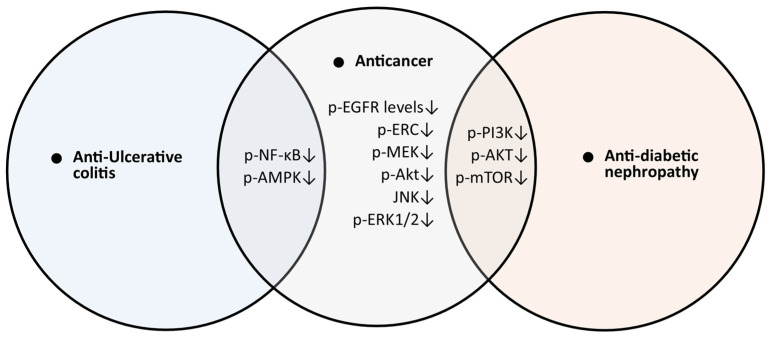
Pathways targeted by trifolirhizin for anticancer activity and other associated diseases. (↓: down-regulation/inhibition).

After analyzing the potential activities of trifolirhizin against the viability, migration, and invasion of the 6–10 B and HK1 cell lines, the mechanism of action of trifolirhizin was studied through transcriptome analysis, which revealed that the PI3K/Akt pathway was among the enriched pathways by the differentially expressed genes identified through a volcano plot and subsequent KEGG pathway analysis. The expression of important genes from the PI3K/Akt pathway was analyzed through Western blot experiment. Trifolirhizin was found to suppress the phosphorylation of both the PI3K and Akt proteins in both 6–10 B and HK1 cells, which suggested that the suppression of PI3K/Akt pathway signaling may be the important mechanism for the anticancer activity of trifolirhizin [10]. The PI3K/Akt signaling pathway is a key regulator in the development of diabetic kidney disease and is known to be targeted by trifolirhizin for anti-DN activity [57]. Similarly, the AMPK/mTOR signaling pathway is also known to be targeted by trifolirhizin for anti-UC activities. The pathways commonly targeted by trifolirhizin in various activities may be considered key targets of trifolirhizin for its multiple pharmacological activities (Figure 2).

Recently, to study the molecular target of trifolirhizin for its reported anticancer activity against nasopharyngeal carcinoma, targets of trifolirhizin were predicted through SuperPred [22]. Protein tyrosine kinase 6 (PTK6) was identified as a direct target of trifolirhizin, which is known to be an important target of various cancers. The nasopharyngeal carcinoma cell line C666-1 was selected to study the anticancer activity in the study, which was found to be inhibited by trifolirhizin in a dose-dependent manner through the CCK-8 assay and EdU staining. Apoptosis was also found to be increased in a dose-dependent manner by trifolirhizin in TUNEL staining. The expression of PTK6, which was predicted to be a direct target of trifolirhizin, was found to be inhibited by trifolirhizin in a WB analysis along with other proteins such as p62, Ki67, and PCNA. In the immunofluorescence analysis, the accumulation of LC3 and the expression of LC3-II/I and Beclin were enhanced by trifolirhizin. In the study, in silico molecular docking analysis also validated the interaction of trifolirhizin with PTK6, visualized through the PyMOL program. The study concluded that trifolirhizin may target PTK6 to induce apoptosis and an anticancer effect. Still, these findings are not validated in in vivo studies, which are suggested to be conducted in the future [22].

#### 5.11.2. In Vivo Anticancer Activities of Trifolirhizin

Intraperitoneal injection of MKN45 cells was used to create tumors in BALB/C nude mice to study cancer activity. After 21 days of the treatment, the weight of the tumors in the treatment groups of all doses was significantly smaller than the control group. Additionally, an immunohistochemistry study revealed a significant decrease in the levels of Ki67-positive cells, which further highlighted the anti-proliferative effect of trifolirhizin, and an increase in cleaved caspase-3-positive cells, which strengthened the trifolirhizin apoptotic activity in the in vivo condition [6].

Later, a cancer model of mice was studied after an in vitro study on colorectal cancer cell lines. SW620 cells were subcutaneously injected into the right flanks of C57BL/6 mice to develop a cancer model [7]. Trifolirhizin (10 mg/kg once in three days) was provided to mice for 21 days, and the Xenograft tumor size in the mice was found to be significantly smaller than in the control group, which suggested the antitumor activity of trifolirhizin the in vivo condition. Hematoxylin and eosin (H&E) and TUNEL staining of tumor tissue showed damage to tumor cells caused by trifolirhizin. Further, in an immuno-staining analysis, trifolirhizin treatment suppressed p-mTOR-positive cells and increased the number of Atg5-, Atg7-, and p-AMPK-positive cells in the tumor.

Finally, the study concluded that trifolirhizin can activate the AMPK/mTOR signaling pathway to cause autophagy and also promote autophagy through the caspase-mediated extrinsic apoptosis pathway. The researchers endorsed trifolirhizin as a promising candidate against colorectal cancer in further studies [7].

After identifying the potential activities of trifolirhizin against the viability, migration, and invasion of human nasopharyngeal carcinoma cell lines (6–10 B and HK1 cells) in vivo, a study was conducted on tumor-bearing nude mice created through the injection of human nasopharyngeal carcinoma cell lines [10]. A significant reduction in weight and volume was observed in the tumors associated with both 6–10 B and HK1 cells (Table 2). A histopathology study was conducted on tissues from the tumor and normal tissues from the kidney and liver through HE staining. The microscopy revealed damage to tumor tissues, while normal tissues from the kidney and liver were unaffected by trifolirhizin, which suggested that trifolirhizin can safely cause an antitumor effect in mice [10].

### 5.12. Wound-Healing Effects of Trifolirhizin

Inspired by the traditional usage of plants and plant products for wound healing in the literature [58,59], a study observed the significant wound-healing activity of acetate extract prepared from the roots of *Ononis spinose*. The most active fraction was used to identify the components important for the wound-healing activity of the extract. Five important phytochemicals including trifolirhizin were studied through the inhibiting assay against enzymes targeted for wound healing such as hyaluronidase, collagenase, and elastase enzymes. The enzyme inhibition activities of trifolirhizin were 28.45 ± 1.96, 27.84 ± 0.72, and 21.53 ± 1.66% for hyaluronidase, collagenase, and elastase enzymes, respectively [21] (Table 1). A relatively low inhibition activity suggested the role of other active compounds may also be important for the wound-healing activities of the extract.

## 6. Gaps and Future Direction

Initially, the pharmacological studies conducted on trifolirhizin were inspired by the known medicinal properties of the traditional medicinal plants in which it is present. Different in vitro, in vivo, and ex vivo experiments have shown various pharmacological activities of trifolirhizin, which make it an important candidate against important diseases. However, different pharmacological activities have associated challenges to overcome. The anti-inflammatory activity of trifolirhizin is associated with its protective effect on different organs such as the liver, kidney, colon, and bone, which also support the multiple pharmacological properties of trifolirhizin (Figure 1).

The protective effect of trifolirhizin against CCl_4_ toxicity in rats was observed through an improvement in important parameters such as the serum level of transaminase enzymes, ALP, the total bilirubin level in serum, and NP-SH in the liver. In a single study, the limited analysis of the molecular mechanisms behind the protective activity was reported; thus, more studies considering the molecular mechanisms the behind protective effect of trifolirhizin are suggested to establish its hepatoprotective activity in further research. Similarly, positive effects of trifolirhizin on the colon in DSS-induced colitis in terms of an improvement in DAI, colon length, and body weight were observed in an in vivo study. The role of the gut microbiome in UC for diagnosis, prognosis, the prediction of therapy outcomes, and as a therapeutic strategy is emerging [60]. Thus, the effect of trifolirhizin on the gut microbiome for anti-UC activities is also suggested to be studied through in vivo experiments before clinical studies [42,60].

The protective effect of trifolirhizin on bone loss was studied in different in vitro and in vivo experiments, which was found to be effective against both ovariectomy-induced and inflammation-induced bone loss. The role of anti-inflammation and the target pathways of trifolirhizin were also revealed in the studies. These important target pathways targeted for bone loss were the MAPK-NFATc1 and NF-κB-MAPK signaling pathways, and the inflammatory factor IL-1β was found to be suppressed in both in vitro and in vivo experiments. Notably, a positive control group was absent in the reported study; hence, in future studies, it is suggested that a positive control group may be added [13]. The study mainly focused on the protective effects of trifolirhizin on bone loss. Importantly, the role of trifolirhizin in the curative treatment of bone loss due to inflammation needs to be analyzed in further studies to strengthen the candidature of trifolirhizin as a therapeutic.

The kidney protective activity of trifolirhizin was studied in diabetic nephropathy in db/db mice. The study also analyzed the important target pathway, i.e., the PI3K/AKT/mTOR pathway, and the role of the antioxidant effect of trifolirhizin in kidney protective activities. However, it is the only study that showed the kidney protective activity of trifolirhizin. Thus, more studies considering different models for kidney injuries are suggested to be conducted in the future to establish trifolirhizin as a kidney-protective therapeutic candidate. Importantly, the PI3K/AKT/mTOR pathway targeted by trifolirhizin is associated with other important diseases, including neurodegenerative disorders, autoimmune diseases, obesity, and diabetes [61]. Thus, studies analyzing the effects of trifolirhizin against these diseases may be conducted in the near future.

Limited antibacterial studies have been conducted for trifolirhizin. However, an in silico study supported its antibacterial activity against the important antibacterial target FtsZ, which is important in pathogenic bacteria [28]. In addition, the effective antibacterial activity of trifolirhizin was found against *Helicobacter pylori*, which was comparable with ampicillin in a study [27]. Therefore, studies against other pathogenic bacteria are strongly suggested to utilize and develop the antibacterial potential of trifolirhizin.

In the search for an active phytoconstituent in the herbal medicine ASHMI™, the antiasthma activity of trifolirhizin through the inhibition of ASM contraction was identified. Trifolirhizin also relaxed precontracted ASM, which again supports its antiasthma activity [4]. The antiasthma effects of trifolirhizin were also found to be effective in a neonatal rat model in terms of elevating inflammation and damage to lung tissues [34]. However, the NF-κB signaling pathway may provide anti-inflammatory-based protection against asthma [34]. It was suggested that a further mechanistic study may be conducted in the near future to develop trifolirhizin as a new candidate for asthma by modulating ASM contraction [4].

The skin-whitening potential of trifolirhizin was observed through its tyrosinase inhibition activity. Additionally, the skin-whitening activity of trifolirhizin was also supported by its ability to reduce the production of intracellular melanin, which was increased due to an inducer. Still, in vivo studies would be required and are suggested in future studies to establish the skin-whitening potential of trifolirhizin.

Activities such as antiplatelet aggregation and the estrogenic effect were found to be effective and much higher than those of the comparable compound macckian [31]. These activities were identified in a single study; thus, more animal studies are suggested to be pursued to verify these activities before clinical studies.

The anticancer activity of trifolirhizin can be considered as one of the most important pharmacological properties, as it was observed against various types of cancers including lung, gastric, ovarian, nasopharyngeal, hepatocellular carcinoma, and colorectal cancer [5,6,7,8,9,10]. Notably, in several studies, the anti-proliferative activity of trifolirhizin was found to be specific to cancer cells, and normal cells were found to be safe or mildly affected, which highlighted its safety profile and again emphasized its candidature as an anticancer drug candidate [5,6,10]. The target pathways in most of these studies were also analyzed to decipher molecular mechanisms and optimize the anticancer activity of trifolirhizin. Important anticancer pathways such as EGFR-MAPK, PI3K/Akt, AMPK/mTOR, ERK, and AKT were found to be targeted by trifolirhizin. These targets are considered core targets and are associated with various types of cancers, which explains the basis of the anticancer activity of trifolirhizin against different types of cancer. Some of these pathways were also found to be targeted in other activities of trifolirhizin, such as anti-DN and anti-UC (Figure 2). It is known that anti-inflammatory activity can also contribute to anticancer activity.

Successful work has been performed to study the anticancer targets of trifolirhizin. However, limited research has been conducted to study the probable off-targets of trifolirhizin, which is suggested in future studies to determine the safety and efficacy of trifolirhizin.

In a nasopharyngeal cancer study, a relatively high dose was used for anticancer effects, which suggests the requirement of further research to optimize the doses while minimizing the side effects [10]. Overall, the anticancer activities of trifolirhizin are prospective and are strongly suggested to be considered in clinical studies after safety experiments.

Largely, trifolirhizin can be considered as a potential compound for various pharmacological properties. However, according to its properties, its development is in different stages. It can be used in further studies through in vitro, in vivo, and, finally, clinical experiments to develop it against important diseases.

## Figures and Tables

**Table 1:** Pharmacological activities of trifolirhizin observed in in vitro studies.

Sr. No.	Activity	Model and Dose	Method	Result	Ref
1	Anti-inflammatory activity	LPS-induced inflammation in the J774A.1 macrophage of the mouse	QRTPCR to study the mRNA expression of tumor necrosis factor (TNF-α) and interleukin-6 (IL-6)	TNF-α ↓ and IL-6 ↓	[8]
			ELISAQRTPCR to study the mRNA expression of tumor necrosis factor (TNF-α)	TNF-α ↓	
			WB to study the protein expression of COX-2	COX-2 ↓	
2	Wound-healing	Inhibition assay of enzymes at 50 and 100 μg/mL	Enzymes hyaluronidase, collagenase, and elastase enzymes	Percentage inhibition (at 100 μg/mL) was 28.45 ± 1.96, 27.84 ± 0.72, and 21.53 ± 1.66 for hyaluronidase ↓, collagenase ↓, and elastase enzymes ↓, respectively	[21]
3	Skin-whitening	Treated with different concentrations (1–500 μg/mL)	Tyrosinase inhibition activity	IC_50_: 506.77 ± 4.94 μM	[20]
		B16 melanoma cells treated with different concentrations (12.5, 25, and 50 μM)	B16 cells induced with IBMX	IC_50_: 36 μM	
4	Protect bone loss	MC3T3-E1 cells were used to study osteogenesis in the study	ALP activity and staining assays	ALP ↑	[15]
			RT-qPCR	ColI ↑, bone sialoprotein Bsp ↑, and Alp ↑	
			Detection of F-actin polymerization in the cells; phalloidin staining	F-actin polymerization ↑	
			Cell migration through Matrigel-coated membranes	Cell migration ↑	
			ARS staining to study mineralization by calcium deposition	Mineralization ↑	
			WB	RUNX2 ↑, β-catenin ↑, p-Smad1/5/8 ↑, p-JNK ↑, p-GSK3β ↑, and p-β-catenin ↓	
			Marker in the immunofluorescence assay	The accumulation of RUNX2 ↑ (in the nucleus)	
		BMM extracted from the bone marrow cavity of the femur and tibia of C57BL/6J and RANKL used as a stimulator Dose: 10, 20, and 40 μM	Cell staining to detect TRAP activity	Significant reduction in the formation of multinucleated osteoclasts was observed at all doses (10, 20, and 40 μM) used in the study	[14]
			Western blot analysis	NFATc1 ↓	
			RT-qPCR	ACP5 ↓, ATc1 ↓, DC-STAMP ↓, MMP9 ↓, V-ATPase-D2 ↓, and CTSK ↓ were found to be downregulated in a dose-dependent manner in the treatment groups	
			Bone resorption assay	In the bone resorption analysis, trifolirhizin treatment (at 20 and 40 μM) significantly decreased absorption area	
5	Anticancer	Human leukemia cells (HL-60 cells)	Viable cell number estimated by the trypan blue dye exclusion method	Proliferation of HL-60 cells ↓	[5]
			Analysis of morphological changes through microscopic observation	Apoptotic bodies and fragmentation of genomic DNA were observed in the treated cells	
		A2780 ovarian cancer cells (5–250 μM)	MTT assay	Proliferation of A2780 ovarian cancer cells ↓ (at 50 μM or more)	[8]
		H23 lung cancer cells (5–250 μM)	MTT assay	Proliferation of H23 lung cancer cells ↓ (at 250 μM)	
		MKN45 cells was 20, 30, and 40 μg/mL	MTT assay	Proliferation of MKN45 cells ↓ (IC50: 33.27 ± 2.06 μg/mL)	[6]
			Flow cytometry Hoechst staining TUNEL staining and Annexin V/PI assay	Increased the number of apoptotic cells, cell shrinkage, nuclear fragmentation, and chromatin compaction of apoptosis accumulation in the G2/M phase in MKN45 cells	
			The JC-10 assay	Normal cells ↓ and apoptotic cells ↑ (20–40 μg/mL trifolirhizin treatment)	
			Western blot analysis	p-EGFR levels ↓, cyclin B ↓, Cdc2 ↓, p-ERC ↓, p-MEK ↓, p-P38 ↑, P53 ↑, c-Myc ↑, caspase-9 ↑, and caspase-3 ↑	
		HCT116 and SW620 cells	Immuno-blotting assay of two autophagy marker proteins, LC-3 and p62/SQSTM-1	LC-3B-I ↑ and LC-3B-II ↑	[7]
			CCK-8 assay	Cell viability ↓	
			Transmission electron microscopy	Autophagic vacuole ↑	
			AdmCherry-GFP-LC3B fluorescent assay	Autophagosomes ↑ and autophagolysosomes ↑	
			Flow cytometry analysis and TUNEL staining	Long-term growth of both cell lines ↓	
			Western blot	Cleaved caspase-3 ↑, cleaved caspase-8 ↑, cleaved poly ADP-ribose polymerase ↑, p-AMPK ↑, and p-mTOR ↓	
		Human nasopharyngeal carcinoma cell lines (6–10 B and HK1)	CCK-8 assays	Cell viability of 6–10 B cells ↓ (IC_50_: 83.67 ± 1.70 μmol/L at 72 h) Cell viability of HK1cells ↓ (IC_50_: 33.21 ± 1.40 μmol/L at 72 h)	[10]
			Flow cytometry analysis through PI staining	Nasopharyngeal cancer cells at G0/G1 phase	
			Scratch assay to study cell migration	6–10 B and HK1 cell migration ↓	
			Transwell assays	Invaded cell numbers ↓ in both 6–10 B and HK1 cells	
			RNA-seq analysis followed by volcano plot and KEGG pathway analysis	The PI/Akt pathway was among the enriched pathways by the differentially expressed genes	
			WB	Phosphorylation of both PI3K and Akt proteins ↓ (in both 6–10 B and HK1 cells)	
		MHCC97H, MHCC97L, and HepG2 cells (12.5, 25, 50, and 100 μg/mL)	MTT assay	IC_50_: 143.1 μg/mL (alone) IC_50_: 1.5 ± 0.06 0.7 ± 0.17 μg/M (in combination with SF (100 μg/mL)) IC_50_: 104.2 μg/mL (alone) IC_50_: 0.7 ± 0.17 μg/M (in combination with SF (100 μg/mL)) IC_50_: >60 μg/mL (alone) IC_50_: 8.4 ± 0.54 μg/M (in combination with SF (100 μg/mL))	[9]
			Microscopy using DAPI Annexin-V FITC/PI analysis using flow cytometry (50.0 μg/mL, 22.4 μM) and SF (2.0 μM)	Nucleus fragmentation ↑ and apoptotic body formation ↑ Percentage of apoptotic cells significantly increased by 5.0-fold for SF-induced apoptosis in comparison to the single treatment of SF	
			Cell cycle assay	Similarly, the combination of compound 17 with SF arrested MHCC97H cells in the G1 phase	
			JC-1 assay	ΔΨm ↓	
			WB	Cyclin D1 ↓ and cyclin B1 ↓, leaved- caspase-3 ↑ cleaved caspase-9 ↑, Bax/Bcl2 ↑, JNK ↓, P38 ↓, p-ERK1/2 ↓, and p-AKT ↓	
		Nasopharyngeal carcinoma cell lines (C666-1) (0, 0.005, 0.01, 0.02, 0.04, 0.08, 0.16, 0.4, 0.8, and 2 mg/mL)	CCK-8 assay and EdU staining	Viability of C666-1 cells ↓	[22]
			TUNEL staining	Apoptosis of C666-1 cells ↑	
			WB	PTK6 ↓, LC3-II/I ↑, Beclin1 ↑, p62 ↓, Ki67 ↓, and PCNA ↓	
			RT-qPCR	PTK6 ↓	
			Immunofluorescence (IF)	LC3 accumulation ↑, levels of LC3-II/I ↑, and Beclin1 ↑	

↑: up-regulation; ↓: down-regulation/inhibition; BMM: bone marrow macrophage; CCK-8: Cell Counting Kit-8; EdU: 5-ethynyl-2′-deoxyuridine; RANKL: receptor activator of nuclear factor-κB ligand; RT-qPCR: real-time quantitative polymerase chain reaction; IBMX: 3-isobutyl-1-methylxanthine; COX-2: cyclooxygenase-2; TNF-α: tumor necrosis factor α; IL-6: interleukin-6; LPS: lipopolysaccharide; TUNEL: terminal deoxynucleotidyl transferase-mediated dUTP-fluorescein nick end labeling; MTT, 3-(4,5-dimethylthiazol-2-yl)-2,5-diphenyltetrazolium bromide; SF: sorafenib; WB: Western blot.

**Table 2:** Pharmacological activities of trifolirhizin observed in in vivo and ex vivo studies.

Sr. No.	Activity	Animal Model and Dose	Method	Results	Reference
1	Hepatoprotective activity	Wistar rats and 7.5 mg/kg (20.7 μmol/kg) 5 days before CCl_4_ administration and continued until the end of the experiment.	Carbon tetrachloride (CCl_4_)-induced liver toxicity, and the levels of liver enzymes in serum were studied	SGOT ↓, SGPT ↓, ALP ↓, and total bilirubin ↓	[31]
			Non-protein sulfhydryl groups in the liver (wet weight)	Non-protein sulfhydryl groups ↑	
2	Antiplatelet aggregation activity	Blood was collected from Wistar rats via cardiac puncture; 400 and 800 μg/mL concentrations were used in the study.	Aggregation of platelets was caused by ADP	Aggregation of platelets ↓	[31]
3	Estrogenic activity	Female Wistar rat model; 20 mg/kg body weight.	Weights of the uteri in control and treated animals to the whole animal weight were also calculated (positive control 17*β*-estradiol)	Uterine weight ↑	[31]
4	Anti-inflammatory activity	Wistar rat paw edema as a model and 4.5 mg/kg dose.	Carrageenan was used to induce edema; indomethacin was used as a positive drug	Paw edema ↓ ^#^ (35%)	[31]
5	Protect against bone loss	Wild-type (WT) C57BL/6J mice; ovariectomy was executed 10 and 20 mg/kg by intraperitoneal injection for 6 weeks.	μCT scanning analysis of tibia and histological assessments	Bone loss ↓ Number of TRAP-positive osteoclasts ↓	[14]
		Male C57BL/6J mice; 5 and 10 mg/kg injected periosteum in the midline of the skull on days 2, 4, 6, and 8.	LPS-induced osteolysis of cranial cap bone analyzed through μCT scanning	Bone loss ↓, BV/TV ↑, and bone destruction area ↓	[13]
			Histological analysis	Number of TRAP-positive osteoclasts ↓, IL-1β-positive area in of cranial cap bone ↓	
6	Anticancer	BALB/C nude mice; dose: 1–3 mg/kg for 21 days of treatment.	Intraperitoneal injection of MKN45 cells to induce tumors in mice	Tumor weight ↓	[32]
			Immunohistochemistry	Ki67-positive cells ↓ and cleaved caspase-3-positive cells ↑	
		BALB/C mice; dose: 10 mg/kg once every three days for 21 days.	Xenograft tumor studies	Size of tumor ↓	[7]
			Hematoxylin and eosin (H&E) and TUNEL staining	Tumor tissue showed the damage of tumor cells	
			WB to study expression	Cleaved caspase-3 ↑, cleaved caspase-8 ↑, p-AMPK ↑, p-mTOR ↓, Atg5 ↑, and Atg7 ↑	
		Male nude mice; dose: administered every other day for 14 days at a dose of 40 mg/kg.	Xenograft tumor model 6–10 B and HK1 cells injected subcutaneously into the right axilla of nude mice	Tumor size and weight ↓	[10]
			HE staining	Tumor tissue showed damage to tumor cells	
7	Anti-UC	C57BL/6 mice; dose: intraperitoneally injected trifolirhizin (12.5, 25, 50 mg/kg) for one time.	Mice received DSS treatment for 7 days with 1.5% DSS in the drinking water	Body weight ↑ and length of the colon ↑	[33]
			Quantitative reverse transcription (qRT)-polymerase chain reaction (PCR).	TNF-α ↓, IL-6 ↓, IL-1β ↓, NLRP3 ↓, caspase 1 ↓, and ASC ↓	
			ELISA	The protein expression of TNF-α ↓, IL-6 ↓, IL-1β ↓, IL-17 ↓, IL-10 ↑, IgM ↑, IgA ↓, and IgG ↓ (colon tissue)	
			WB	p-nuclear factor (NF)-κB/NF-κB ↓, RORγt protein ↓, Foxp3 ↑, NLRP3 ↓, caspase 1 ↓ and ASC ↓, IL-1β ↓, p-AMPK/AMPK ↓, and TXNIP ↑	
			Flow cytometry analysis of cells from mesenteric lymph nodes and the spleen	Th17 (CD4+ IL17+) cells ↓ Treg (CD4+ CD25+ Foxp3+) cells ↑	
			Immunofluorescence staining	NLRP3 expression ↓	
8	Anti-diabetic nephropathy	Male db/db mice; trifolirhizin (0, 12.5, 25 and 50 mg/kg); 3 weeks.	Histological analysis of renal tissues was performed by H & E staining	Body and renal weight ↓, fasting blood glucose ↓, renal injury ↓	[16]
			TUNEL staining of renal tissues	Apoptosis ↓	
			ELISA	BUN ↓, creatinine ↓ MDA ↓, and SOD ↑	
			WB of renal tissues	LC3II ↑, Beclin1 ↑, p62 ↓, p-PI3K/PI3K ↓, p-AKT/AKT ↓, and p-mTOR/mTOR ↓	
			DHE staining of renal tissues	ROS ↓	
9	Antiasthma	Six-week-old BALB/c asthmatic mouse model induced by albumin sensitization and challenge.	ASM contraction in tracheal rings was induced by acetylcholine	ASM contraction ↓	[4]
		Neonatal pups of SD rats; asthmatic mouse model induced by albumin sensitization.	Histopathology	Tissue damage ↓, aggregation of inflammatory cells ↓, edema in pulmonary tissues ↓, and histological scores ↓	[34]
			Immunohistochemistry	*Muc5AC* ↓ and *Muc5B* ↓ genes (the lungs) TNF-α ↓ and ICAM-1 ↓, IL-4 ↓, IL-5 ↓, and IL-13 ↓ (in BALF)	
			WB	IκBα protein expression ↑	

↑: upregulation; ↓: downregulation/inhibition; ^#^: insignificant reduction; SGOT: serum glutamate oxaloacetate transaminase; SGPT: serum glutamate pyruvate transaminase; ALP: alkaline phosphatase; BV/TV: bone volume/tissue volume; μCT: micro-computed tomography; DHE: dihydroethidium; DSS: dextran sulfate sodium; ROS: reactive oxygen species; MDA: malondialdehyde; SOD: superoxide dismutase; BUN: blood urea nitrogen; ASM: airway smooth muscle contraction; BW: body weight.

## Data Availability

Data are contained within this article.
